# Depth of neutrophil mobilization stratifies survival in ST-elevation myocardial infarction

**DOI:** 10.1038/s44161-026-00836-0

**Published:** 2026-07-03

**Authors:** Mathis Richter, Jessica von Göwels, Maximilian Fähndrich, Christian Lipgens Fernandez, Katharina Grohn, Marius Welzel, Dennis Schwarz, Alexander Lang, Amin Polzin, Sara Reinartz Groba, Luca Farinola, Susmita Ghosh, Jan-Niklas Heming, Hanna Aleth, Anastassia Akhalkatsi, Kian Marjani, Nicole Rübsamen, Tobias Radecke, Frank Rosenbauer, Albert Sickmann, André Karch, Alexander Zarbock, Jens Minnerup, Jürgen Sindermann, Alexander Bender, Jan Rossaint, Steffen Ormanns, Matthias Gunzer, Mariano Malamud, Rainer Kaiser, Kami Pekayvaz, Dominik Heider, Holger Reinecke, Lena Makowski, Christian Jung, Carlos Silvestre-Roig, Malte Kelm, Norbert Gerdes, Raphael Chevre, Stefan A. Lange, Oliver Soehnlein

**Affiliations:** 1 Institute of Experimental Pathology (ExPat), Center of Molecular Biology of Inflammation (ZMBE), University of Münster, Münster, Germany; 2 Department of Cardiology I, University Hospital Münster, Münster, Germany; 3 Institute of Medical Informatics, University of Münster, Münster, Germany; 4 Department of Cardiology, Pulmonology and Vascular Medicine, University Hospital and Medical Faculty, Heinrich-Heine University, Düsseldorf, Germany; 5 Cardiovascular Research Institute Düsseldorf (CARID), Heinrich-Heine University, Düsseldorf, Germany; 6 Department of Internal Medicine B, University of Münster, Münster, Germany; 7 Leibniz-Institut für Analytische Wissenschaften ISAS – e.V., Dortmund, Germany; 8 Department of Anaesthesiology, Intensive Care and Pain Medicine, University Hospital Münster, Münster, Germany; 9 Department of Anaesthesiology and Intensive Care Medicine, University Hospital Essen, University Duisburg-Essen, Essen, Germany; 10 Institute of Molecular Tumour Biology, University of Münster, Münster, Germany; 11 Department of Medicine I, University Hospital, LMU Munich, Munich, Germany; 12 DZHK (German Centre for Cardiovascular Research), Munich, Germany; 13 Institute of Epidemiology and Social Medicine, University of Münster, Münster, Germany; 14 Department of Neurology, University Hospital Schleswig-Holstein, Lübeck, Germany; 15 DZHK (German Centre for Cardiovascular Research), Lübeck, Germany; 16 Institute of General Pathology, Medical University Innsbruck, Innsbruck, Austria; 17 Innpath Institute of Pathology, Tirol Kliniken, Innsbruck, Austria; 18 Institute for experimental Immunology and Imaging, University Hospital Essen, University of Duisburg-Essen, Essen, Germany

**Keywords:** Myocardial infarction, Predictive markers

## Abstract

Systemic inflammatory responses shape clinical outcomes in acute cardiovascular disease. Because of their functional plasticity and rapid turnover, neutrophils have emerged as dynamic indicators of inflammatory stress. Here we profile the appearance of distinct neutrophil maturation stages in patients with ST-elevation myocardial infarction, heart failure and stroke. Our data reveal the mobilization of immature neutrophils in all groups; however, patients with ST-elevation myocardial infarction exhibited the most pronounced engagement of this response, including the appearance of CD16^low^CD10^neg^ preneutrophils (preNeus), the final mitotic neutrophil progenitor, which was associated with disease outcome. Plasma cytokine profiling identified a coordinated inflammatory signature associated with preNeu mobilization, consistent with emergency granulopoiesis. PreNeus were identifiable as immature granulocytes in routine blood count analysis, where they predicted 30-day mortality better than established biomarkers in two clinical cohorts. Immature granulocytes also remained independently associated with survival in multivariate models incorporating risk factors, enabling the immediate identification of vulnerable patients with ST-elevation myocardial infarction upon hospital admission.

## Abstract (web summary)

Richter et al. show that the mobilization of neutrophil progenitors assessed from routine blood tests can predict 30-day mortality in patients following ST-elevation myocardial infarction and enable rapid risk stratification.

## Main

Almost a century ago, Smith and Goodrich reported that the ‘neutrophilic partition’—a pronounced and persistent left shift—identified adverse outcome following coronary occlusion, suggesting the possibility that the appearance of neutrophil developmental states in blood indicates clinically meaningful biology^1^. Yet, over the following decades neutrophils were considered mere bystanders in cardiovascular inflammation. In the past decade, however, this paradigm has shifted as numerous studies have unveiled causal roles for neutrophils across several inflammatory cardiovascular pathologies^2,3^. Such analyses, however, typically consider the global neutrophil population and do not discriminate between neutrophil maturation and activation stages^4^.

Neutrophils have the highest turnover of any cell in the human body^5^. They develop in the bone marrow from specialized stem and progenitor cells, which are collectively referred to as the mitotic pool. Neutrophils develop in a stepwise manner from granulocyte–monocyte progenitors. The granulopoietic trajectory continues with proneutrophil 1 cells, which transition to proneutrophil 2 cells and further to proliferative preneutrophils (preNeus), ultimately giving rise to post-mitotic immature and mature neutrophils^6,7^. This maturation is a highly organized process characterized by the stepwise acquisition of maturation markers (for example, CD16 and CD10) and loss of retention markers (for example, CXCR4, CD49d).

In the steady state, the circulating neutrophil compartment is almost exclusively comprised of mature cells^4^; however, in situations of inflammation and subsequent emergency granulopoiesis^8^, immature cells are mobilized to the bloodstream. Rather than representing a binary switch, accumulating experimental and clinical evidence indicates that emergency granulopoiesis can proceed with varying degrees of strength, driving the release of progressively more immature neutrophil progenitors as inflammatory stress escalates^9–12^. This phenomenon, historically referred to as a ‘left shift’, reflects the increased release of different stages of neutrophil maturation from the bone marrow to meet the elevated peripheral demand. Granulopoiesis is regulated by growth factors and cytokines including granulocyte-colony stimulating factor, interleukin (IL)-6 and IL-1 family members, suggesting that the systemic inflammatory response directly affects the depth of neutrophil mobilization^13–15^.

Acute cardiovascular syndromes are characterized by profound and heterogeneous inflammatory responses. However, it remains unclear whether this biological variability is captured by the deployment of different neutrophil developmental states to the circulation. Because circulating neutrophil levels are particularly early and sensitive responders to insults, we here hypothesize and show that the appearance of neutrophil maturation stages in the bloodstream, in particular the depth of mobilization of progressively more immature neutrophils to the circulation, may function as a biological sentinel of systemic inflammatory stress and disease severity, enabling risk stratification in acute cardiovascular disease.

## Results

### Graded depth of neutrophil mobilization across cardiovascular disease entities

**Fig. 1:**
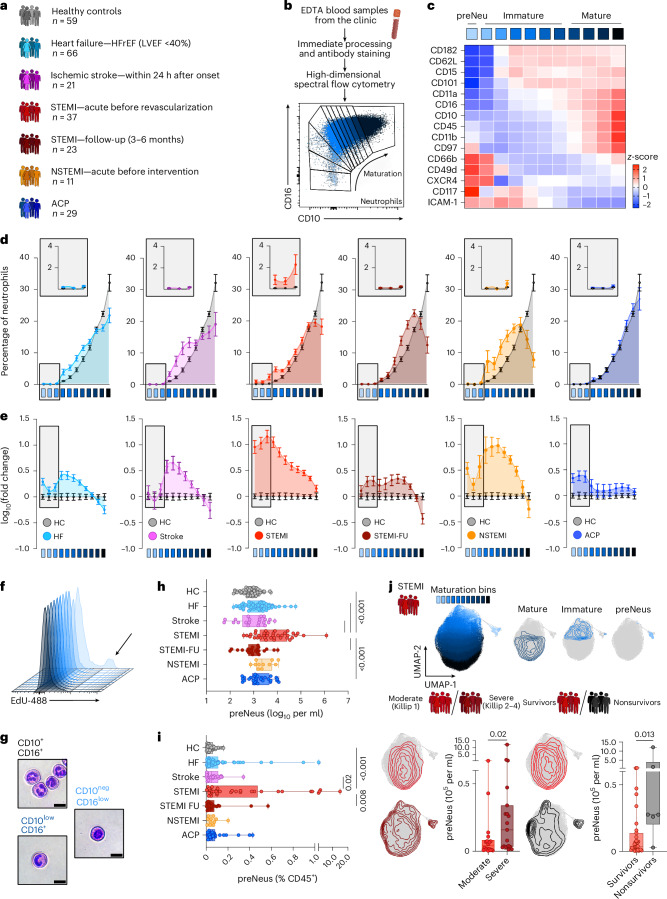
Neutrophil maturation landscapes across cardiovascular disease states. **a**, Patient groups. **b**, Study design. **c**, The *z*-score-normalized surface marker expression in different neutrophil maturation bins. **d,e**, Relative abundance (**d**) and log_10_-fold change in absolute neutrophil maturation stage abundance (**e**) in HC (gray), HF (light blue), stroke (purple), STEMI (red), STEMI follow-up (STEMI-FU; dark red), NSTEMI (yellow) and ACP without evidence of epicardial coronary obstruction (blue) versus HC (gray). Insets represent magnified immature bins 1–3. Mean ± s.e.m. **f**, Proliferation assessed by ex vivo EdU incorporation and detection using flow cytometry. **g**, Giemsa staining of FACS-purified cells of different maturation stages. Scale bar: 10 µm. **h**,**i**, Abundance of preNeus in blood from different cohorts. One-way analysis of variance (ANOVA) with Dunnett’s test (**h**) or Kruskal–Wallis with Dunn’s test (**i**) comparing STEMI with all other groups. Numbers of patients are given in **a**. **j**, UMAP of STEMI neutrophils (top) and absolute preNeu counts across different patient subgroups (bottom) (based on CD10, CD16, CD66b, CD11b, CD49d, CD62L, CXCR2 and CXCR4). Two-tailed Mann–Whitney test. *n* = 18 moderate, 18 severe (1 STEMI patient without Killip score), 31 survivors and 6 nonsurvivors. Box plots indicate median and IQR (25th–75th percentile); whiskers extend to minimum and maximum values with individual data points shown. HFrEF, HF with reduced ejection fraction; LVEF, left ventricular ejection fraction. Source data

To determine whether distinct cardiovascular disease states engage emergency granulopoiesis at different depths, we profiled the appearance of neutrophil maturation states in the blood of patients with heart failure (HF), ischemic stroke, non-ST-elevation myocardial infarction (NSTEMI) and STEMI before revascularization. In the STEMI cohort, we included a follow-up blood-drawing 3–6 months after STEMI. Furthermore, we included an acute chest pain (ACP) cohort without evidence of epicardial coronary obstruction (Fig. 1a). EDTA blood samples of patients and healthy controls (HC) were processed immediately after collection and stained for high-dimensional spectral flow cytometry to resolve neutrophil maturation along the CD10 and CD16 axes (Fig. 1b).

**Extended Data Fig. 1:**
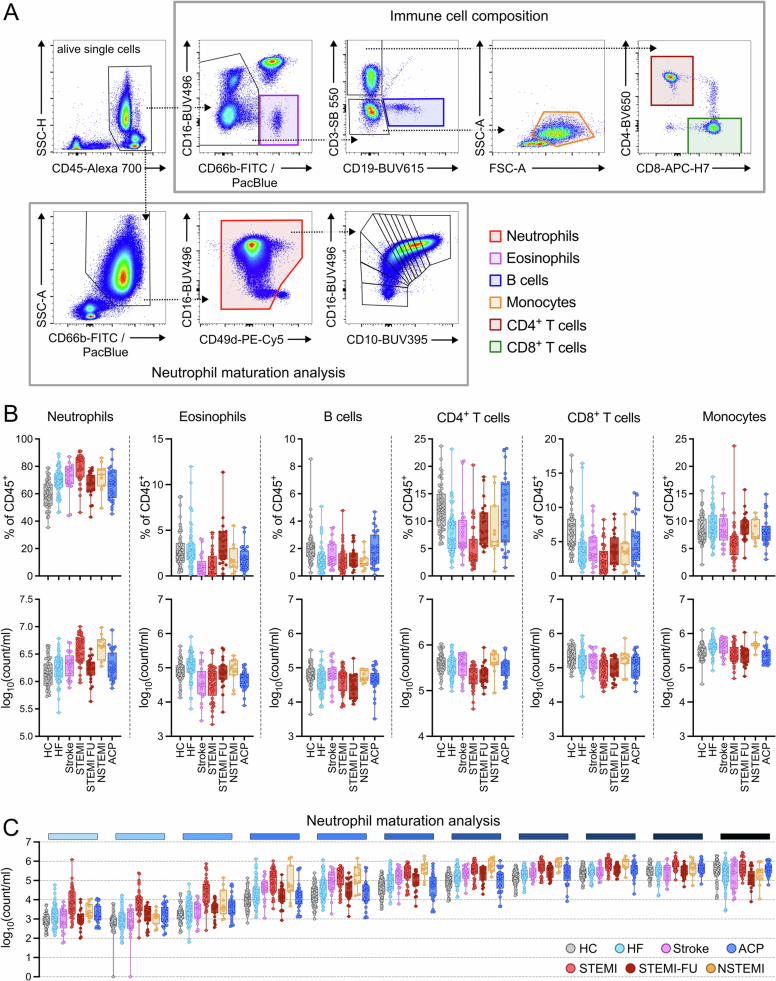
Cardiovascular inflammation leads to neutrophilia. (**A**) Flow cytometry gating strategy. (**B**) Relative (top) or absolute (bottom) blood counts of indicated leukocyte subsets. (**C**) Absolute abundance of neutrophil maturation stages across groups. n = 59 for HC, n = 66 for HF, n = 21 for stroke, n = 37 for STEMI; n = 23 STEMI follow-up (FU), n = 11 NSTEMI, n = 29 ACP; HC, health control; HF, heart failure; STEMI, ST-elevation myocardial infarction. HC = healthy control, HF = heart failure, (N)STEMI = (non-) ST-segment elevation myocardial infarction, ACP = acute chest pain. Box plots indicate median and IQR (25th–75th percentile); whiskers extend to minimum and maximum values with individual data points shown. Source data

Based on the expression of CD10 and CD16, we subdivided circulating neutrophils into 11 bins thus covering a continuous neutrophil maturation trajectory that allowed interrogation of a progressive and dynamic remodeling of the neutrophil population along maturation (Fig. 1c). In line with emergency granulopoiesis, all cardiovascular disease cohorts exhibited a left shift toward immature neutrophil states compared to HCs; this shift was minimal in patients with ACP and was largely resolved at follow-up in patients with STEMI (Fig. 1d,e and Extended Data Fig. 1). Importantly, the composition of this left shift differed markedly across disease states: patients with HF and stroke predominantly expanded late immature neutrophils, patients with NSTEMI showed a deeper displacement toward earlier developmental stages, and acute STEMI was characterized by a profound shift extending to the most immature compartments (Fig. 1d,e). Notably, patients presenting with ACP in the absence of myocardial ischemia showed only minimal engagement of these shallow compartments, indicating that acute pain and stress alone are unlikely to drive deep neutrophil mobilization. Likewise, the abundance of very immature neutrophil stages normalized 3–6 months after STEMI, demonstrating that this response represents a transient, injury-linked inflammatory state rather than a persistent alteration of hematopoiesis.

Interestingly, the most immature cluster exhibited overt expression of the retention molecules CXCR4 and CD49d and incorporated the thymidine analog 5-ethynyl-2′-deoxyuridine (EdU) ex vivo, thus reflecting the last proliferating neutrophil progenitor; that is, the preNeu (Fig. 1f). In agreement with this, fluorescence-activated cell sorting (FACS) followed by Giemsa staining revealed a segmented nucleus in CD16^high^CD10^high^ mature neutrophils, a banded nucleus in CD16^int^CD10^low^ immature neutrophils and a round nucleus in CD16^low^CD10^neg^ preNeus (Fig. 1g). Therefore, we refer to these very immature cells as preNeus.

Having established the identity of this most immature compartment, we next quantified circulating preNeus across cohorts. Patients with STEMI exhibited the highest preNeu abundance, whereas those with HF, stroke and NSTEMI showed lower levels, and preNeu counts normalized at follow-up after STEMI (Fig. 1h,i), consistent with a graded depth of neutrophil mobilization across cardiovascular disease entities.

In the STEMI cohort, circulating preNeu levels varied widely between individuals (Fig. 1h,i). Neutrophils from this cohort were visualized along the maturation continuum (Uniform Manifold Approximation and Projection for Dimension Reduction (UMAP); Fig. 1j). Using the Killip classification^16^, a classical clinical score of HF severity following STEMI associated with mortality, patients in Killip classes 2–4 exhibited pronounced enrichment of the most immature compartments, including preNeus, compared with those in Killip class 1. Likewise, analysis stratified by 30-day survival revealed that nonsurvivors showed a strong shift toward these deeply immature regions relative to survivors, indicating that progressively deeper immaturity in the neutrophil mobilization accompanies worsening clinical status and predicts short-term mortality. Together, these findings define the depth of neutrophil mobilization as a graded biological program that reflects the magnitude of the inflammatory response, especially after STEMI.

### Inflammatory programs are associated with neutrophil mobilization depth

**Fig. 2:**
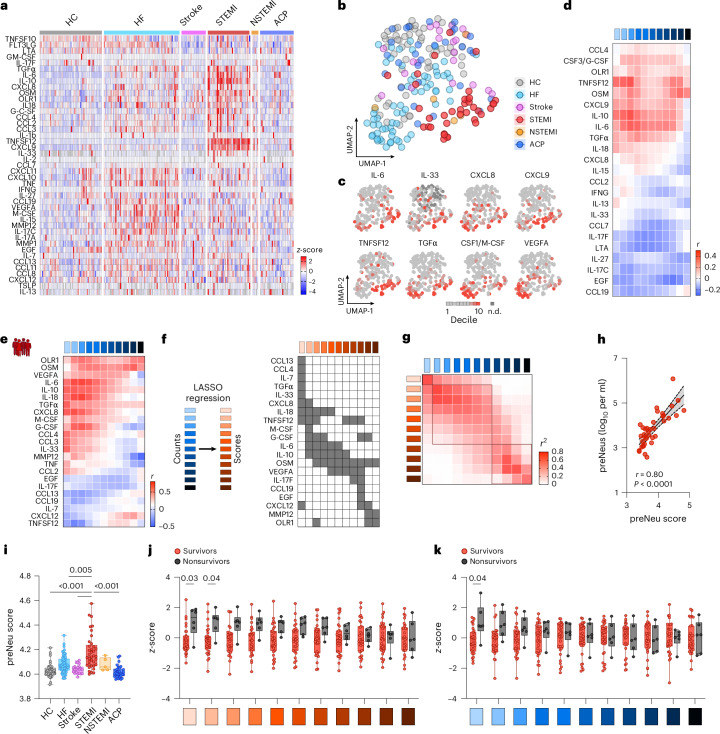
Cytokine programs scale with neutrophil mobilization depth in STEMI. **a**, Heatmap of targeted plasma proteomics of 43 cytokines and inflammatory mediators measured across the indicated cohorts. *n* = 54 HC, *n* = 66 HF, *n* = 21 stroke, *n* = 37 STEMI, *n* = 5 NSTEMI, *n* = 29 ACP. The *z*-scores per analyte are shown and measurements under the detection limit are displayed in gray. **b**,**c**, UMAP of plasma proteomics data colored by cohort (**b**) and abundance of selected cytokines displayed as deciles (**c**) highlighting a distinct inflammatory profile in STEMI compared with the other cohorts. **d**, Pearson correlation analysis of cytokine concentrations with the abundance of individual neutrophil maturation compartments across all samples. Cytokines that are significantly correlated (*P* < 0.05) with at least one neutrophil maturation stage are shown. **e**, Corresponding cytokine–neutrophil Pearson correlation analysis restricted to patients with STEMI. Cytokines that are significantly correlated (*P* < 0.05) with at least one neutrophil maturation stage are shown. **f**, Cytokine scores derived by LASSO regression predicting the abundance of each neutrophil maturation bin in STEMI. **g**, Pearson correlation analysis of cytokine scores with neutrophil maturation compartments. **h**, Linear relationship between preNeu-specific cytokine score and circulating preNeu counts. Linear regression analysis. Shaded areas indicate 95% confidence bands. **i**, PreNeu cytokine score calculated across disease cohorts. Kruskal–Wallis with Dunn’s test comparing STEMI with all other groups. *n* = 54 HC, *n* = 66 HF, *n* = 21 stroke, *n* = 37 STEMI, *n* = 5 NSTEMI, *n* = 29 ACP. **j**,**k**, The *z*-scores of cytokine scores (**j**) and corresponding neutrophil maturation compartment counts (**k**) stratified by 30-day survival in patients with STEMI. Two-way ANOVA with Šídák’s test. *n* = 31 survivors, *n* = 6 nonsurvivors. Box plots indicate median and IQR (25th*–*75th percentile); whiskers extend to minimum and maximum values with individual data points shown. n.d., not detected. Source data

To define the inflammatory programs associated with different depths of neutrophil mobilization, we performed targeted plasma proteomics in the cohorts analyzed in Fig. 1, quantifying 43 cytokines and growth factors (Fig. 2a). Unsupervised clustering revealed a distinct inflammatory profile in patients with STEMI compared with patients with HF and stroke. This was characterized by elevated levels of cytokines linked to myocardial injury and tissue stress, including IL-6, tumor necrosis factor ligand superfamily member 12 and IL-33 (Fig. 2b,c). This cytokine signature distinguished STEMI from the other cardiovascular disease entities and is consistent with a STEMI-specific inflammatory program.

To link the mobilized neutrophil maturation depth to systemic inflammation, we examined how circulating cytokines varied with the abundance of each neutrophil developmental stage across all measured samples. This analysis revealed that progressively deeper neutrophil mobilization was accompanied by increasingly strong inflammatory signatures, indicating that emergency granulopoiesis leading to the mobilization of progressively more immature neutrophils is tuned by the magnitude and composition of the inflammatory response in these patients (Fig. 2d).

To resolve these relationships specifically in acute myocardial infarction, we restricted the cytokine-maturation analyses exclusively to the STEMI cohort. Even in this single disease state, mobilization of progressively deeper neutrophil maturation stages was associated with increasingly strong and structured inflammatory signatures (Fig. 2e), indicating that variation in mobilized neutrophil maturation depth among patients with STEMI reflects graded differences in their inflammatory milieu. In patients with STEMI, we next asked whether different stages of neutrophil mobilization are associated with distinct inflammatory programs rather than a single uniform cytokine response. To address this, we used least absolute shrinkage and selection operator (LASSO) regression, a statistical method that builds a predictive model while automatically selecting the most relevant parameters, to derive cytokine scores that best predicted the abundance of each neutrophil maturation bin (Fig. 2f). This revealed that early and intermediate immature neutrophil compartments were linked to overlapping cytokine scores including classical inflammatory mediators such as IL-6, IL-10, oncostatin M, granulocyte-colony stimulating factor and vascular endothelial growth factor A. By contrast, the cytokine signature associated with preNeus was compositionally distinct and included mediators such as IL-33, transforming growth factor alpha and CXCL8. Consistent with this, correlation of all cytokine scores with all maturation bins showed a largely graded increase across immature and mature neutrophil compartments, whereas the preNeu score displayed a partially distinct pattern that differed from the patterns observed for earlier stages of neutrophil release (Fig. 2g). Thus, although increasing inflammatory activity progressively drives the appearance of immature neutrophils in blood, the emergence of preNeus marks a qualitatively different inflammatory state associated with the deepest level of neutrophil mobilization in STEMI. Notably, the preNeu-specific cytokine score showed a strong linear relationship with circulating preNeu counts (Fig. 2h), indicating that this distinct inflammatory program quantitatively reflects the depth of emergency granulopoiesis in individual patients with STEMI.

We next examined how the preNeu-associated inflammatory program and neutrophil maturation states relate to disease context and clinical outcome. Across the different disease entities analyzed, the preNeu cytokine score reached its highest levels in patients with STEMI, consistent with the deepest engagement of emergency granulopoiesis in this setting (Fig. 2i).

**Extended Data Fig. 2:**
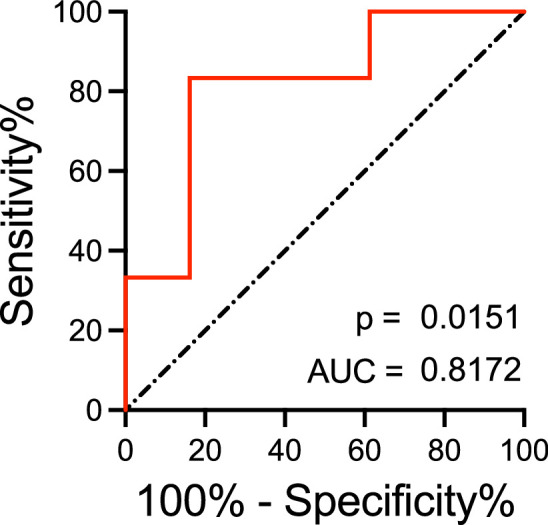
PreNeu counts are predictive of survival. Receiver-operator characteristic (ROC) analysis of preNeu counts on admission as a predictor of survival. n = 37. Source data

In the STEMI cohort, cytokine scores associated with multiple immature neutrophil compartments were elevated in nonsurvivors, reflecting a broadly amplified inflammatory response (Fig. 2j). By contrast, when examining circulating cell counts, only preNeus were selectively increased in nonsurvivors (Fig. 2k and Extended Data Fig. 2).

These findings indicate that a fatal outcome after STEMI is accompanied by broad inflammatory activation and that the ensuing mobilization of the deepest neutrophil progenitor compartments provides an applicable readout of mortality risk.

### Immature granulocytes capture deep neutrophil mobilization in routine blood counts

**Fig. 3:**
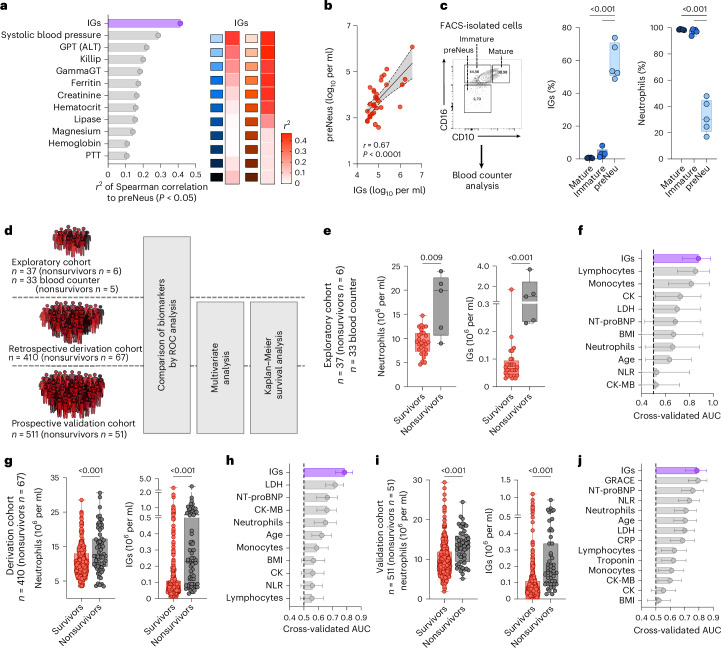
PreNeus are detected as IGs and predict short-term mortality. **a**, Spearman correlation of preNeus with clinical parameters (left) and correlation of IGs with different neutrophil maturation states and cytokine scores (right). **b**, Linear regression of preNeus identified by flow cytometry with IGs from automated blood counter analysis. *n* = 33. **c**, FACS of neutrophil subsets followed by automated blood counter-based identification of IGs and neutrophils. One-way ANOVA with Holm-Šídák’s test. Sorting and analysis strategies are detailed in Extended Data Fig. 4. *n* = 5 sorted blood samples. **d**, Analyzed clinical cohorts: the exploratory cohort analyzed in Figs. 1 and 2, a retrospective derivation cohort and a prospective validation cohort. **e**,**g**,**i**, IG and neutrophil counts on admission in the exploratory cohort (**e**), derivation cohort (**g**) and validation cohort (**i**). Two-tailed Mann–Whitney test. **f**,**h**,**j**, AUC values represent discrimination estimated from out-of-fold predictions obtained by fivefold cross-validation in the exploratory cohort (**f**), derivation cohort (**h**) and validation cohort (**j**). Error bars indicate 95% confidence intervals. Box plots indicate median and IQR (25th–75th percentile); whiskers extend to minimum and maximum values with individual data points shown. ALT, alanine aminotransferase; BMI, body mass index; CRP, C-reactive protein; gammaGT, gamma-glutamyl transferase; GPT, glutamate-pyruvate transaminase; NLR, neutrophil–lymphocyte ratio; PTT, partial thromboplastin time; ROC, receiver operating characteristic. Source data

**Extended Data Fig. 3:**
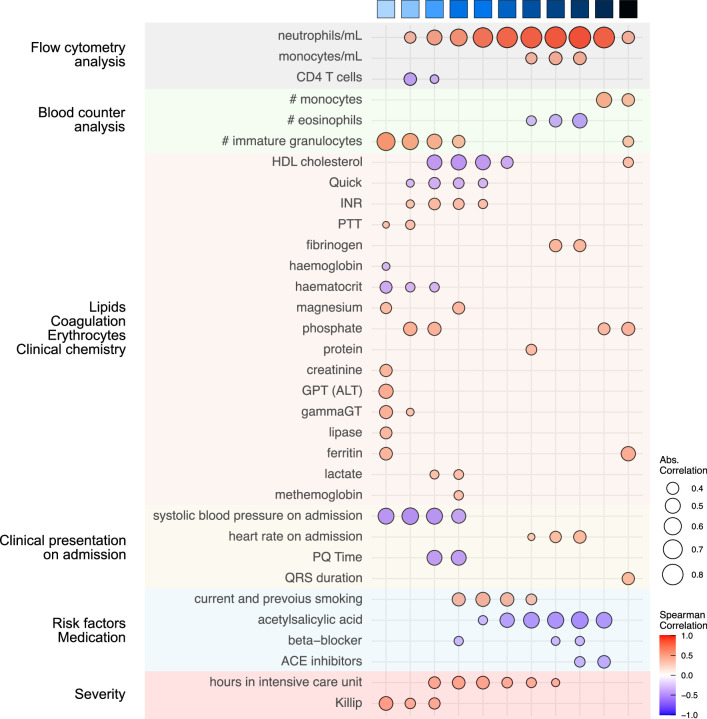
Correlation analysis of maturation stages against clinical parameters. Two-tailed spearman correlations of absolute counts of neutrophil maturation bins with clinical parameters including blood counter analysis, clinical chemistry, clinical presentation, patient characteristics and severity/outcome. Significant correlations (p < 0.05) are shown. Source data

The use of spectral flow cytometry to identify and quantify circulating preNeus in an emergency setting represents a notable challenge in routine clinical practice. Consequently, we systematically correlated the abundance of flow cytometry-defined neutrophil maturation stages with routine clinical parameters obtained at the time of intervention. This analysis of neutrophil maturation with blood parameters gathered in the clinic around the time of the intervention led to the identification of the parameter ‘immature granulocytes’ (IGs), reported by standard differential blood cell counts, as the single parameter showing the strongest correlation with circulating preNeus (Fig. 3a and Extended Data Fig. 3). Clinical blood counter tests were conducted on the majority of patients with STEMI (33 of 37). IGs correlated most strongly with the most immature neutrophil compartments and showed robust associations with cytokine scores linked to immature neutrophil stages, consistent with IGs capturing the deepest levels of granulopoietic mobilization (Fig. 3a).

**Extended Data Fig. 4:**
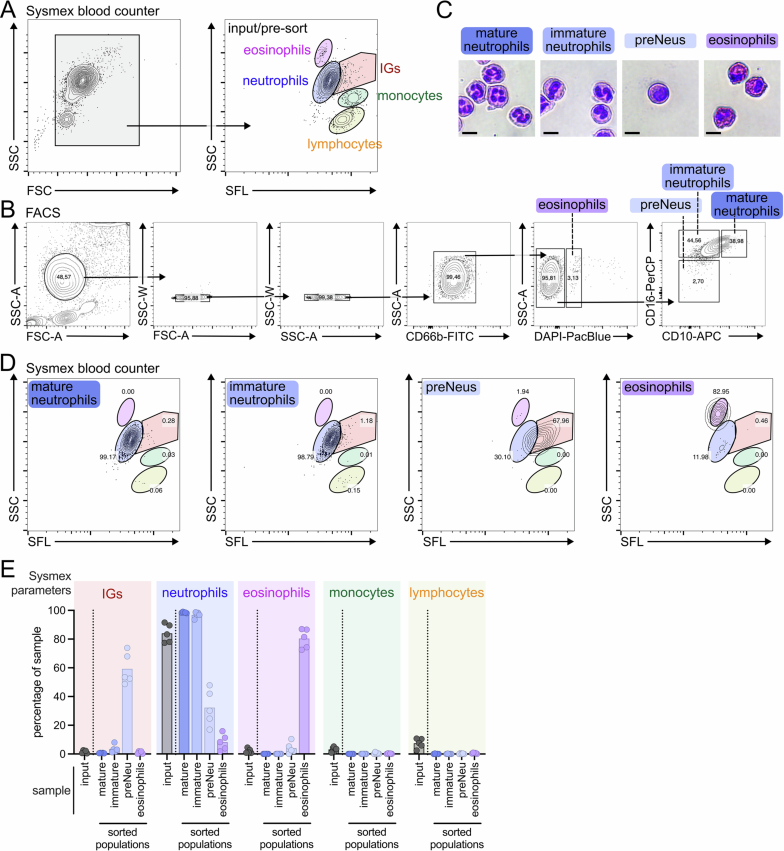
PreNeus but not mature or immature neutrophils or eosinophils are identified as immature granulocytes (IGs) in automated blood counter analysis. (**A**) FlowJo gating strategy to identify immune cell populations from Sysmex blood counter analyses based on scatter characteristics (Side Scatter Signal (SSC)) and DNA/RNA content (Side Fluorescence Signal (SFL)) in line with the automatic annotation of different immune cells in whole blood by the machine. (**B**) FACS sorting strategy to isolate mature neutrophils, immature neutrophils, preNeus and eosinophils. (**C**) Giemsa staining of isolated cell populations. Scale bar, 10 µm. (**D**) FlowJo gating of isolated cell types. (**E**) Percentage of identified cells from Sysmex blood counter analysis in input/pre-sort and FACS-isolated cell populations. n = 5 sorted blood samples. Source data

PreNeus identified by spectral flow cytometry analysis correlated with the IGs from the automated blood counter analysis (Fig. 3b), supporting the use of IGs as a scalable surrogate for deep neutrophil mobilization. To directly test whether preNeus identified by flow cytometry are contained in the IGs in the clinical blood counts, we isolated mature neutrophils, immature neutrophils and preNeus by FACS based on CD66b, CD10 and CD16 expression profiles, and subsequently analyzed each population separately using a clinical blood counter (Fig. 3c and Extended Data Fig. 4). Only sorted CD10^neg^CD16^low^ preNeus but not mature and immature neutrophils were classified as IGs in the blood counter analysis (Fig. 3c and Extended Data Fig. 4d,e). Of note, eosinophils sorted from these samples were not identified as IGs (Extended Data Fig. 4c–e). These findings establish that IGs specifically reflect the presence of the most immature circulating neutrophil progenitors rather than general neutrophilia.

Having identified a readily accessible parameter for approximating circulating preNeus, we next assessed its prognostic performance in post hoc analyses across three independent STEMI cohorts: (1) the exploratory cohort that we analyzed by flow cytometry (Extended Data Table 1); (2) a retrospective derivation cohort of patients with STEMI admitted to University Hospital Münster from 2019 to 2025 and receiving a differential blood cell count within the first 24 h after admission (*n* = 410), 67 of whom died in the first 30 days after STEMI (Extended Data Table 2); and (3) a larger NCT-listed, prospective validation cohort of patients with STEMI receiving contemporary guideline-directed therapy with complete revascularization (SYSTEMI)^17^, including 511 patients 51 of whom died within 30 days (Fig. 3d and Extended Data Table 3).

Corroborating the results of our flow cytometric analyses, IG levels were markedly elevated in nonsurvivors in all three cohorts (Fig. 3e,g,i). Interestingly, cross-validated area under the receiver operating characteristic curve (AUC) analysis demonstrated that IGs showed greater discrimination than all other single-parameter biomarkers for the prediction of 30-day survival, including risk factors such as age or body mass index, and blood parameters such as neutrophil–lymphocyte ratio, creatine kinase (CK), CK-MB, lactate dehydrogenase (LDH), N-terminal pro–B-type natriuretic peptide (NT-proBNP) and C-reactive protein or troponin where available (Fig. 3f,h,j). This performance of IGs was observed reproducibly in the exploratory, derivation and prospective validation cohorts, indicating that IGs represent a robust single-parameter predictor of short-term mortality after STEMI. Interestingly, IGs also displayed an AUC comparable with the Global Registry of Acute Coronary Events (GRACE) score (v.1.0 for in-house mortality)^18^, a risk score for survival after acute coronary syndrome comprised of eight parameters, in the validation cohort (Fig. 3j).

**Extended Data Fig. 5:**
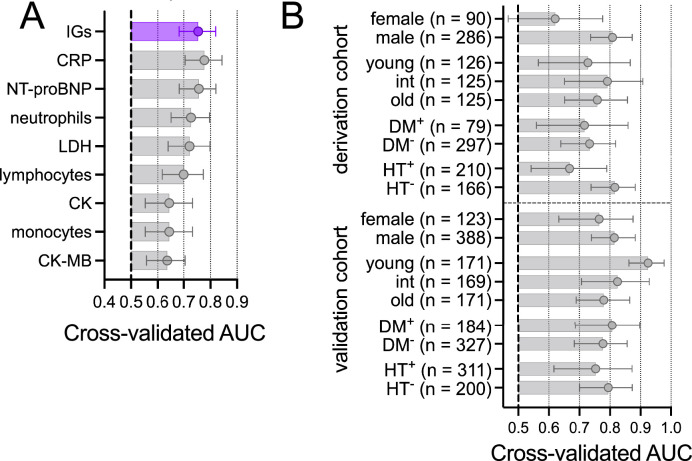
Immature granulocytes (IGs) remain predictive of mortality across subgroups and on day 1 post STEMI. (**A**) Area under the curve (AUC) values represent discrimination estimated from out-of-fold (OOF) predictions obtained by 5-fold cross-validation for markers of survival on day 1 after STEMI. Error: 95% CI. n = 378 survivors, n = 34 non-survivors. (**B**) AUC for IGs as a biomarker of survival in different subgroups. Error: 95% CI. DM = Diabetes mellitus, HT = hypertension. Source data

**Extended Data Fig. 6:**
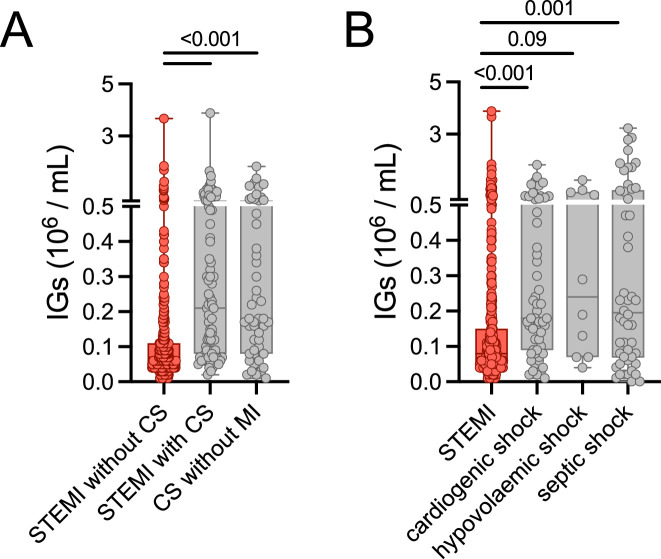
Immature granulocytes (IGs) in STEMI with or without cardiogenic shock (CS) and CS without MI and different aetiologies of shock. (**A**) Retrospective data analysis of patients with the main diagnosis STEMI (ICD-10 code I21.0–I21.3) with (n = 85) or without (n = 325) cardiogenic shock (ICD-10 code R57.0) as a secondary diagnosis and patients with a CS main diagnosis and no MI (ICD-10 code I21; n = 50) secondary diagnosis at the University clinic Münster. Kruskal-Wallis with Dunn’s test. (**B**) Retrospective data analysis of patients with the main diagnosis STEMI (ICD-10 code I21.0 – I21.3, n = 410), cardiogenic shock (ICD-10 code R57.0; n = 56), hypovolaemic shock (ICD-10 code R57.1; n = 10) or septic shock (ICD-10 code R57.2; n = 50) at the University clinic Münster. Kruskal-Wallis with Dunn’s test. Box plots indicate median and IQR (25th–75th percentile); whiskers extend to minimum and maximum values with individual data points shown. Source data

Of note, in the validation cohort, we additionally compared biomarkers 1 day after hospital admission. This analysis revealed that IGs also had one of the highest cross-validated AUCs at this delayed time point (Extended Data Fig. 5a). Further analyses stratified by parameters such as sex, age, diabetes mellitus and hypertension revealed that IGs performed consistently across all subgroups except for females in the smaller retrospective derivation cohort only (Extended Data Fig. 5b). High IG counts were associated with a severe clinical presentation indicated by a cardiogenic shock (Extended Data Fig. 6a) and patients with shock of different etiologies independent of MI showed comparably high IG counts compared to severe MI patients (Extended Data Fig. 6).

### Immature granulocytes provide independent prognostic value beyond established risk models

**Fig. 4:**
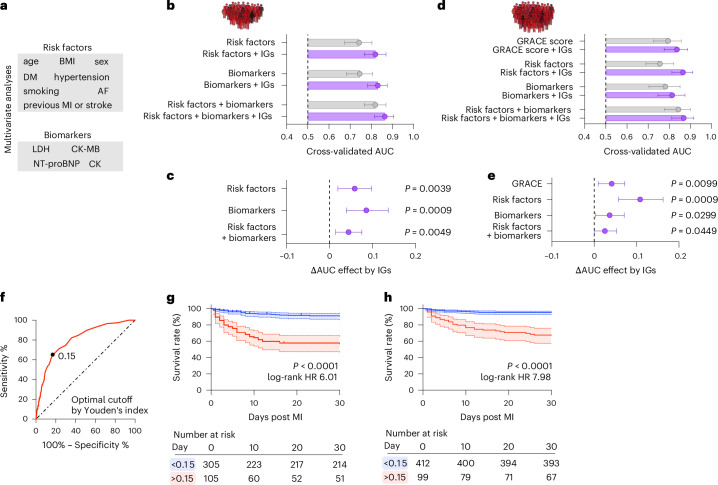
IGs improve multivariable risk prediction and stratify survival in STEMI. **a**, Clinical covariates and laboratory biomarkers included in multivariable risk prediction models. **b**,**c**, Cross-validated out-of-fold AUC (**b**) and ΔAUC (**c**) for models incorporating clinical risk factors, laboratory biomarkers or their combination with or without IGs in the retrospective derivation cohort. **d**,**e**, Corresponding cross-validated out-of-fold AUC (**d**) and ΔAUC (**e**) analyses in the prospective validation cohort, including the GRACE score. *n* = 343 survivors and *n* = 67 nonsurvivors (**b**,**c**); *n* = 460 survivors, *n* = 51 nonsurvivors (**d**,**e**). **f**, Determination of the optimal IG cutoff using Youden’s index across pooled cohorts. **g**,**h**, Kaplan–Meier survival curves stratified by the IG cutoff in the derivation cohort (**g**) and validation cohort (**h**); censored observations are indicated by tick marks. Error bars indicate 95% confidence intervals. AF, atrial fibrillation; DM, diabetes mellitus; HR, hazard ratio. Source data

To assess whether IGs provide independent prognostic information beyond established clinical variables and laboratory markers, we performed multivariate risk prediction analyses in the retrospective derivation cohort and the prospective validation cohort. The clinical covariates and shared biomarkers included in these models are summarized in Fig. 4a. In the derivation cohort, models incorporating clinical risk factors alone or established laboratory biomarkers alone showed only moderate discrimination for 30-day mortality (Fig. 4b). Addition of IGs increased AUCs across all model classes. Analysis of the difference in out-of-fold AUC (ΔAUC) analysis demonstrated that the inclusion of IGs resulted in consistent gains in discrimination, including when added to models combining clinical risk factors and biomarkers (Fig. 4c).

We next evaluated the robustness of these findings in the prospective validation cohort. In this cohort, models based on the GRACE score, clinical risk factors or laboratory biomarkers showed moderate discrimination for 30-day mortality (Fig. 4d). The addition of IGs was associated with higher AUCs across all model classes. ΔAUC analysis confirmed consistent incremental gains in discriminatory performance upon inclusion of IGs, when added to the GRACE score or when added to clinical risk factors and biomarkers (Fig. 4e).

To define a clinically practical IG threshold for risk stratification, we determined an optimal cutoff using Youden’s index across both cohorts, thereby leveraging the largest available STEMI population to obtain a robust estimate suitable for future studies (Fig. 4f). This analysis identified an IG threshold of 0.15 × 10^6^ cells ml^−1^.

**Extended Data Fig. 7:**
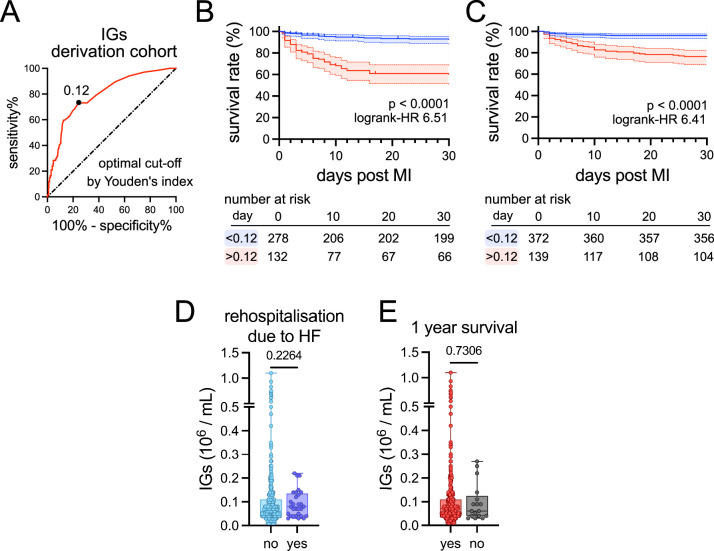
Immature granulocytes (IGs) stratify short-term survival but are not predicting long-term outcome after STEMI. (**A**) Determination of the optimal IG cut-off using Youden’s index in the derivation cohort. (**B**/**C**) Kaplan–Meier survival curves stratified by the IG cut-off in the derivation cohort (**B**) and validation cohort (**C**); censored observations are indicated by tick marks. Error bars indicate 95% confidence intervals. IG counts of 30-day STEMI survivors stratified by (**D**) rehospitalization due to heart failure (n = 431 non-hospitalised, n = 29 hospitalised) or (**E**) 1-year survival (n = 443 survivors, n = 17 non-survivors). Two-tailed Mann-Whitney test. HF = heart failure. Source data

When patients in the derivation cohort were stratified according to this prespecified cutoff, patients with IG^high^ exhibited a substantially higher risk of death within 30 days compared with those with IG^low^ (log-rank hazard ratio = 6.01), as illustrated by Kaplan–Meier survival analysis (Fig. 4g). Separation of survival curves occurred early after admission, consistent with IGs capturing prognostically relevant information at presentation. We next examined the performance of the same IG threshold in the independent prospective validation cohort. Application of this cutoff reproducibly stratified patients into high- and low-risk groups, with an almost eight times higher risk of dying in the IG^high^ group (hazard ratio = 7.98) (Fig. 4h). Calculation of the cutoff based only on the derivation cohort and applying to both cohorts resulted in similar results (Extended Data Fig. 7a). Of note, IGs did not predict long-term survival or hospitalization for heart failure among 30-day survivors (Extended Data Fig. 7b).

Together, these findings show that the depth of mobilized neutrophil immaturity is encoded by distinct inflammatory programs and can be captured by IGs as a scalable marker to stratify short-term mortality risk in STEMI.

## Discussion

Clinical observations made as early as 1935 suggested profound changes in the maturation state of circulating neutrophils during acute myocardial infarction^1^, yet their biological importance and clinical implications beyond neutrophil numbers^19^ and the neutrophil–lymphocyte ratio^20,21^ remained unresolved for decades. Here we show that these changes in the circulating neutrophil compartment reflect the graded engagement of emergency granulopoiesis, with increasing inflammatory stress associated with the mobilization of progressively more immature neutrophil progenitors into the circulation. By linking this biological continuum to short-term mortality risk and translating it into a routinely available clinical biomarker, our study provides a contemporary framework for interpreting long-standing observations of neutrophil responses in acute cardiac injury.

Inflammation is a key pathogenic driver ensuing upon myocardial ischemia, but immune states that emerge during acute injury and subsequent clinical implications remain poorly defined. Multiomic factor analysis has recently revealed multifaceted rewiring of the immune landscape upon cardiac ischemia^22^. Yet, the complexity of this analytical approach prohibits its application in an acute clinical setting. Of note, the assessment of neutrophils whose omics features are not readily accessible by routine transcriptomic workflows was not undertaken in this study. Our dedicated neutrophil-centric analyses indicated that blood from patients with stroke, HF and STEMI exhibits nondiscriminating features of emergency granulopoiesis including neutrophilia and the appearance of immature neutrophils in the blood. Immature neutrophils have been linked to adverse outcomes across various conditions including cardiovascular inflammation and infections because of their heightened proinflammatory activity, including reactive oxygen species production and neutrophil extracellular trap release^23,24^. Extending these observations, our data demonstrate that emergency granulopoiesis proceeds with varying depths across patients and disease states, resulting in the graded mobilization of progressively more immature neutrophil stages into the bloodstream. Functional interrogation of neutrophils spanning different maturation states in STEMI may uncover mechanistic links to disease pathophysiology.

Our findings indicate that the appearance of the most immature circulating neutrophil populations, including CD16^low^CD10^neg^ preNeus, reflects the deepest engagement of this granulopoietic response. Given their low frequency in blood and their quick disappearance from the circulation, it is unlikely that these cells play a key pathogenic role during the acute insult. Instead, their presence appears to report the magnitude and composition of the systemic inflammatory response accompanying myocardial infarction. Consistent with this interpretation, neutrophil progenitor abundance was not closely associated with markers of infarct size such as CK-MB, troponin or LDH.

Plasma cytokine profiling revealed that progressively deeper neutrophil mobilization is associated with structured inflammatory programs, with a partially distinct cytokine signature emerging at the deepest level of granulopoiesis. However, mechanisms underlying the differential depth of neutrophil mobilization remain unclear and are likely multifactorial. One potential explanation is differential signaling originating from the heart, either through humoral pathways involving the release of alarmins or through systemic stress responses. Notably, ACP without evidence of epicardial coronary obstruction was not associated with the deep neutrophil mobilization observed in STEMI, arguing against pain perception or sympathetic activation as primary drivers of this response. Intriguingly, while classical inflammatory cytokines correlated with the release of immature neutrophils, the cardiac alarmin IL-33 was specifically associated with mobilization of the most immature neutrophil states.

Retrospective analyses of patients separated by cardiogenic shock revealed that high IG mobilization in STEMI is strongly associated with disease severity and patients with shock of different etiologies showed high IG counts comparable to severe STEMI, indicating that shock is an unspecific driver of IG mobilization. Taken together, our data indicate that the mobilization of neutrophils of varying maturation reflects the magnitude of systemic inflammation and the ensuing stress on the bone marrow, and is not specific to myocardial ischemia per se; however, these multifactorial stress signals to the bone marrow seem to be the strongest in our STEMI cohort among the cohorts analyzed using flow cytometry in this study.

An important aspect not addressed in this study is neutrophil production in the bone marrow or extramedullary sites such as the spleen. It is tempting to speculate that interindividual variability in granulopoiesis—potentially influenced by (epi)genetic factors, alterations in retention molecule expression or changes in hematopoietic niches—may contribute to the mobilization of neutrophils at distinct maturation stages^25^.

Although both STEMI and NSTEMI represent acute myocardial infarction, we observed a shallower depth of neutrophil mobilization and a heterogenous inflammatory phenotype in the plasma in NSTEMI, with a lower abundance of the most immature circulating compartments compared with STEMI. NSTEMI is a clinical heterogenous disease ranging from low-risk to high-risk patients, therefore, larger cohorts are needed in future to assess the full extent of neutrophil responses in this disease. Furthermore, whether preNeu mobilization provides prognostic information in NSTEMI about disease outcome requires systematic investigation in larger, dedicated cohorts stratified to the risk profile. Together, these findings support the notion that deep neutrophil mobilization serves as a quantitative readout of an exaggerated systemic inflammatory state that is closely associated with short-term mortality risk in STEMI.

Importantly, we demonstrate that this biological axis can be captured using IGs, a routinely available parameter in automated blood counts, overcoming the problem of labor- and time-intensive and error-prone analyses using flow cytometry in an acute disease such as STEMI. IGs specifically reflect the presence of the most immature circulating neutrophil stages and have the highest AUCs even when compared with established single-parameter biomarkers in predicting 30-day mortality after STEMI in both clinical cohorts in our study. Beyond single-marker performance, IGs provide independent added prognostic value when combined with clinical risk factors, laboratory biomarkers and the GRACE score, highlighting their potential utility for early risk stratification in routine clinical care. Although newer iterations exist, GRACE v.1.0 for in-house mortality was applied as the only version whose complete algorithm is fully specified in a peer-reviewed publication^18^, ensuring independent reproducibility. GRACE 2.0 and 3.0 were not used because their coefficients are not fully published in citable form^26^ and the latter was derived exclusively non-ST-elevation acute coronary syndrome populations^27,28^. Finally, we defined a cutoff value of 0.15 × 10^6^ IG ml^−1^, which was able to reliably stratify patients with STEMI into high- and low-risk groups in both cohorts (hazard ratio values of 6.01 and 7.98, respectively) using a one-measurement blood count parameter on the day of admission. This could help allocate extended intensive care monitoring to high-risk patients with STEMI. Additional deeper analyses of the clinical and inflammatory state of patients with IG^low^ and IG^high^ could potentially identify druggable targets for new targeted and personalized therapeutic approaches. Notably, circulating preNeus normalized in the STEMI follow-up group. In line with this, IGs did not predict long-term survival or hospitalization for heart failure beyond 30 days, showing that immature neutrophil mobilization is a transient response to acute inflammatory stress rather than a marker of chronic risk.

Several limitations of this study should be acknowledged. First, detailed characterization of neutrophil maturation states by high-dimensional flow cytometry was performed in a relatively small exploratory cohort, reflecting the technical and logistical constraints of applying such analyses in acute clinical settings. Although these findings were supported by larger retrospective and prospective cohorts using IGs as a surrogate marker, future studies with larger flow cytometry-based datasets are important to further refine the cellular responses during acute myocardial injury. Second, although our analyses establish strong associations between deep neutrophil mobilization, inflammatory programs and short-term mortality, they do not comprehensively address causality or the mechanistic signals driving progenitor release from the bone marrow. Finally, the IG cutoff proposed here (0.15 × 10^6^ IG ml^−1^) was derived from pooled cohorts to maximize robustness and should be prospectively evaluated in independent populations and clinical contexts. However, when the cutoff was calculated in the derivation cohort only and then applied to both cohorts, the results were similar, highlighting that IGs are a robust marker by which to stratify the risk profile of patients with STEMI across cohorts.

In summary, our study identifies the maturation depth of mobilized neutrophils as a biologically grounded and clinically accessible axis of risk stratification in patients with STEMI, providing a framework for future studies to explore how inflammatory regulation of myelopoiesis may inform precision risk assessment and therapeutic decision-making in acute cardiovascular disease.

## Methods

### Ethics of flow cytometry patient groups

Informed consent was obtained from healthy donors and patients with the approval of the Ethics Committee of University of Münster (study numbers 2021-424-f-S, 2021-532-f-S, 2023-348-f-S, 2023-489-f-S and 2024-253-f-S). We collected EDTA-anticoagulated blood between 2022 and 2024 at the University Hospital Münster from 37 patients with STEMI before revascularization (median age (interquartile range (IQR)): 59 (50.75–69), 33 male (89%)); 11 patients with NSTEMI before revascularization (median age (IQR): 76 (60.3–87.7), 9 male (82%)); and 29 patients with ACP without troponin dynamics in the emergency department (median age (IQR): 54.5 (47.75–67.25), 19 male (65%)) to assess the potential influence of factors not exclusively associated with myocardial infarction (for example, fear of death, stress, pain). EDTA blood was also collected from 21 patients with ischemic stroke within the first 24 h after onset of symptoms (median age (IQR): 62 (53.5–82.5), 15 male (71%)); 66 patients with HF with reduced left ventricular ejection fraction < 40%; median age (IQR): 68 (59.5–75), 54 male (82%)); and 59 HCs (median age (IQR): 58 (42–63.5), 37 male (%): 37 (63%)). For all cohorts represented in Fig. 1, patients with anemia, acute infectious diseases, acute inflammatory diseases or chronic inflammatory diseases; kidney, liver, hematological or coagulation disorders; malignancies; recent trauma; surgery in the last 6 months; severe bleeding requiring a blood transfusion; hormonal treatment or taking anti-inflammatory drugs, genetic disorders or psychiatric abnormalities; pregnancy and lactation were excluded. Blood was processed immediately afterwards to preserve the acute neutrophil phenotype.

Etiologies for the ACP and HF with reduced ejection fraction cohorts are described in Supplementary Tables 1 and 2.

### Details on derivation STEMI cohort

Anonymized patient information including blood counter analysis, clinical chemistry and survival from patients with STEMI at the University Hospital Münster between 2019 and 2025 was gathered using a data bank query at the Medical Data Integration Center (MeDIC) of the University Münster with the approval of the Ethics Committee of University of Münster (study number 2024-185-f-S). Individuals were identified as patients with STEMI based on their main diagnosis ICD-10 code (I21.0–I21.3). Risk factors were identified by ICD-10 codes of secondary diagnoses: diabetes mellitus (E10/11), smoking (F17.2), hypertension (I10/11), atrial fibrillation (I48), previous MI (I25.2) or stroke (I69). Patients who received a differential blood count within the first 24 h after admission were included.

### Details on validation STEMI cohort (SYSTEMI)

All patients were enrolled in the systemic organ communication in STEMI (SYSTEMI) study (NCT03539133), an open-end prospective cohort study that collects data from patients with STEMI and high-risk patients with NSTEMI treated at the University Hospital Duesseldorf^17^. The study was approved by the ethics committees of the Heinrich-Heine-University in Duesseldorf, Germany (#5961R), and complies with the Declaration of Helsinki. Informed consent was obtained from all enrolled patients included. Blood used for the automated differential blood count was drawn following acute myocardial infarction diagnosis at hospital admission before starting coronary angiography and revascularization. In a subcohort of patients in whom differential blood counts were available, IG dynamics at day 1 were analyzed. Detailed information about the biosampling protocol was previously published^17^. Patients with STEMI admitted to the hospital between 2019 and 2023 for whom automated differential blood counts, including IG count right after percutaneous coronary intervention, were available were included in the study. Following percutaneous coronary intervention, all patients received standard medication and were treated according to European guidelines^29^.

### Retrospective analysis of IG counts in shock patients

Anonymized IG counts from patients with shock identified using the ICD-10 codes R57.0 (cardiogenic shock), R57.1 (hypovolemic shock) or R57.2 (septic shock) as their main diagnosis at the University Hospital Münster between 2019 and 2025 were gathered using a data bank query at the Medical Data Integration Center (MeDIC) of the University Münster with the approval of the Ethics Committee of University of Münster (study number 2024-185-f-S).

### Spectral flow cytometry

For flow cytometric analysis of leukocytes, EDTA blood was incubated with red blood cell lysis buffer (BioLegend) for 20 min on ice for erythrocyte lysis. After washing, cells were centrifuged and resuspended in HBSS (without Mg^2+^ and Ca^2+^, 0.06% BSA, 0.3 mM EDTA) and unwanted Fc receptor-involved staining blocked using Human TruStain FcX (BioLegend). Afterwards, cells were incubated with fluorophore-conjugated antibodies for 20 min on ice in cell staining buffer (BioLegend) before measurement. Antibodies used in the study are listed in Supplementary Table 3.

Data acquisition was performed on a 5L-Cytek Aurora (Cytek Biosciences), and data were analyzed using FlowJo v.10.10.0 (BD Bioscience), Rstudio and GraphPad Prism 10 (GraphPad Software). Doublets were excluded using forward and side scatter characteristics and dead cells using DAPI. Neutrophils were identified as CD66b^+^SSC^high^CD49d^low^ cells, eosinophils as CD66b^+^CD16^low^ cells, B cells as CD66b^−^CD19^+^CD3^−^ cells, T cells as CD66b^−^CD19^−^CD3^+^ cells and further divided into CD4^+^ and CD8^+^ T cells, while monocytes were defined as CD66b^−^CD19^−^CD3^−^SSC^int^FSC^high^ cells in FlowJo (Extended Data Fig. 1a). The UMAP dimensional reduction in Fig. 1 was performed in FlowJo based on neutrophil markers CD10, CD16, CD66b, CD11b, CD49d, CD62L, CXCR2 and CXCR4 and then visualized using R.

### Targeted plasma proteomics

Plasma cytokine profiling was performed by a commercial service provider using the Olink Target 48 Cytokine panel (PEA Technology), following the manufacturer’s protocols. Two analytes were excluded from downstream analyses because of low detection (IL-4) or known regulation of hepatocyte growth factor by heparin, a common treatment of patients with STEMI before admission to the hospital.

Absolute analyte abundances were log_10_(*x* + 1)-transformed for downstream analyses. For UMAP dimensional reduction, data were scaled and left-censored missing values were imputed using a minimal value deterministic imputation.

### LASSO regression

LASSO regression was used to identify cytokines associated with log_10_(*x* + 1)-transformed cell population values. Cytokine measurements were log_10_(*x* + 1)-transformed; missing values were imputed using minimal value deterministic imputation, and all predictors were standardized before model fitting. LASSO regression was performed with a Gaussian error distribution and an L1 penalty using the glmnet package (v.4.1-10) in R (v.4.5.1). The optimal regularization parameter (*λ*) was selected by tenfold cross-validation using the minimum cross-validated error. A cytokine score was calculated for each sample and each maturation stage as the linear predictor from the final model.

### Cross-validated receiver operating characteristic analysis and multivariate analysis

Cross-validated receiver operating characteristic analyses and multivariate analyses were performed in Python (v.3.12.2) using the following packages: NumPy (v.2.0.1), pandas (v.2.2.3), scikit-learn (v.1.8.0), statsmodels (v.0.14.5), patsy (v.1.0.1), SciPy (v.1.15.3) and Matplotlib (v.3.10.6).

Discrimination performance was quantified using out-of-fold AUC derived from fivefold cross-validation. AUCs are depicted as 0.5–1 independent of the directionality.

Multivariate analyses were performed separately in the retrospective derivation and prospective validation cohorts to evaluate the prognostic value of IGs for 30-day mortality and their incremental value beyond established clinical and laboratory predictors. The primary endpoint was 30-day mortality, coded as a binary outcome.

Covariates included clinical confounders (age, body mass index, sex, diabetes, hypertension, smoking status, prior myocardial infarction, prior stroke and atrial fibrillation), acute laboratory biomarkers (CK, CK-MB, LDH, NT-proBNP), and the absolute number of IGs at day 0 (primary biomarker of interest). GRACE score 1.0 was included in the validation cohort based on Granger et al.^18^. Right-skewed biomarkers were clipped to non-negative values and log1p-transformed before modeling. Continuous variables were standardized to mean 0 and standard deviation 1.

#### Prediction modeling and discrimination

Prediction models were fitted using logistic regression with L2 regularization (class_weight=balanced). Model performance was evaluated using fivefold stratified cross-validation, with all preprocessing steps (imputation, transformation and scaling) performed in each training fold and applied to the corresponding test fold to prevent data leakage. Analyses were performed separately in each cohort using the same cross-validation procedure. Missing data were handled using iterative imputation for continuous variables and mode imputation for binary variables. Missingness indicators (NA_variable) were added for predictors with missing values and included as additional covariates. Model discrimination was quantified using the AUC, estimated from out-of-fold predictions. Confidence intervals for the AUC were derived using stratified bootstrap resampling.

#### Added value analyses

To quantify the incremental prognostic value of IG, nested models with and without IG were compared within each cohort. Added value was assessed by ΔAUC. Statistical significance was evaluated using stratified bootstrap resampling (2,000 iterations), resampling events and nonevents separately to obtain 95% confidence intervals and two-sided *P* values for ΔAUC, computed via a sign-test on the bootstrap distribution of ΔAUC.

### Youden’s index cutoff

Youden’s index threshold calculation was performed using the pROC package (v.1.19.0.1) in R (v.4.5.1) on the derivation cohort or combined data set.

### Clinical blood test

The clinical blood tests were performed according to local standard of care within the first 24 h of hospitalization. We used the following parameters: CK, CK-MB, LDH, neutrophils, lymphocytes, monocytes and IGs. Differential blood count analyses were performed in the central clinical laboratories using a Sysmex XN-9000 (University Hospital Düsseldorf), XN-9100 or XN-1000 (University Hospital Münster) (all SYSMEX EUROPE SE).

### Cell sorting of granulocyte subsets and blood counter analysis

EDTA blood was lysed and FcR-blocked before cells were stained using the following cleavable antibodies: anti-CD66b (FITC, Miltenyi Biotec, 1:50), anti-CD10 (APC, Miltenyi Biotec, 1:50) and anti-CD16 (PerCP, Miltenyi Biotec, 1:50). After mature and immature neutrophils, preNeus and eosinophils were sorted using an FACS Aria III (BD), antibodies were cleaved from the sorted cells and the input using REAlease reagent (Miltenyi Biotec) to not interfere with blood counter analysis. Sorted cells were then measured in a Sysmex XN-1000. Because the system is not designed to identify and annotate isolated cell types, we extracted the .fcs files from the machine and performed cell-type identification using sideward scatter characteristics and side fluorescence signal in FlowJo. For this, we placed the gates for neutrophils, monocytes, lymphocytes, eosinophils and IGs based on the whole blood and the automatic annotation of these samples in the machine. This strategy is explained in more detail in Extended Data Fig. 4.

### Giemsa staining

Flow-sorted cell populations were spun onto object slides using a Cellspin III centrifuge (Thermac, 5 min, 270*g*). Cells were fixed in methanol for 7 min and incubated (45 min) with Giemsa stain (Sigma-Aldrich) before imaging.

### Proliferation assay

EDTA blood was lysed and then incubated with EdU (Baseclick, 10 μM) in RPMI medium (Gibco, with 2% fetal calf serum) for 1 h at 37 °C and 5% CO_2_. Cells were then stained using conjugated antibodies before EdU incorporation was detected according to manufacturer instructions (Baseclick) and acquired on a flow cytometer.

### Statistical analyses

Statistical analysis was performed using Python (v.3.12.2) for the AUC analyses and multivariate analyses or GraphPad Prism 10 (GraphPad Software). The indicated statistical tests for continuous variables were performed dependent on normal distribution and equality of standard deviation. Statistical tests indicated in the figure legend were used. Data are displayed as box plots, mean ± s.e.m. or mean ± 95% confidence interval as indicated in the figure legends.

### Declaration of AI-assisted technologies in the writing process

During preparation of this manuscript, the authors used ChatGPT (OpenAI), Claude AI (Anthropic) and DeepL Write (DeepL) to improve English language and readability. After using these tools, the authors reviewed and edited the content as needed and take full responsibility for the content of the publication.

### Reporting summary

Further information on research design is available in the Nature Portfolio Reporting Summary linked to this article.

## Supplementary information

Supplementary Information Supplementary Tables 1–3.

Reporting Summary

## Source data

Source Data Fig. 1 Statistical source data.

Source Data Fig. 2 Statistical source data.

Source Data Fig. 3 Statistical source data.

Source Data Fig. 4 Statistical source data.

Source Data Extended Data Fig. 1 Statistical source data.

Source Data Extended Data Fig. 2 Statistical source data.

Source Data Extended Data Fig. 3 Statistical source data.

Source Data Extended Data Fig. 4 Statistical source data.

Source Data Extended Data Fig. 5 Statistical source data.

Source Data Extended Data Fig. 6 Statistical source data.

Source Data Extended Data Fig. 7 Statistical source data.

## Data Availability

Source data are provided with this paper.

## Extended data

**Extended Data Table 1:** Descriptive parameters of the exploratory cohort (Münster)

Characteristics	STEMI survivors (n = 31)	STEMI non-survivors (n = 6)	p-value (Mann-Whitney/Chi-square)
Age, years, median (IQR)	58 (48 – 68)	62 (60 – 71)	0.16
Male, n (%)	27 (87%)	6 (100%)	0.35
Body-mass-index, kg/m^2^, median (IQR)	26,43 (24,87 – 29,34)	30,75 (23,85 – 33,49)	0.17
Systolic arterial pressure, mmHg, median (IQR)	127 (103 – 139)	81 (76 – 100)	0.006
Diastolic arterial pressure, mmHg, median (IQR)	78 (59 – 85)	64 (32.5 – 66.5)	0.045
Heart rate, bpm, median (IQR)	82 (68 – 96)	84,5 (73.25 – 106.5)	0.61
**Risk factors**			
Diabetes mellitus, n (%)	5 (16%)	3 (50%)	0.07
Current and previous smoking, n (%)	15 (48%)	3 (50%)	0.94
Arterial hypertension, n (%)	11 (35%)	3 (50%)	0.50
Hyperlipoproteinemia, n (%)	16 (52%)	0 (0%)	0.02
Positive family history for MI, n (%)	10 (32%)	0 (0%)	0.10
**Medication**			
Acetylsalicylic acid, n (%)	5 (16%)	1 (17%)	0.97
Clopidogrel/P2Y inhibitors, n (%)	1 (3%)	0 (0%)	0.66
Marcumar/Thrombin inhibitors, n (%)	1 (3%)	0 (0%)	0.66
Beta-blocker, n (%)	5 (16%)	3 (50%)	0.07
ACE inhibitors, n (%)	4 (13%)	2 (33%)	0.21
Diuretic drugs, n (%)	1 (3%)	0 (0%)	0.66
Ca antagonist, n (%)	0 (0%)	1 (17%)	0.02
AT-1 blocker, n (%)	5 (16%)	1 (17%)	0.97
Lipid-lowering drugs, n (%)	5 (16%)	1 (17%)	0.97
Aldosterone antagonist, n (%)	1 (3%)	0 (0%)	0.66
Antidiabetic drugs, n (%)	3 (10%)	2 (33%)	0.12
**Patient history**			
Myocardial infarction, n (%)	4 (13%)	1 (17%)	0.81
Percutaneous coronary intervention, n (%)	4 (13%)	1 (17%)	0.81
Apoplexy, n (%)	2 (6%)	2 (33%)	0.05
Peripheral artery disease, n (%)	2 (6%)	1 (17%)	0.40

**Extended Data Table 2:** Descriptive parameters of the retrospective derivation cohort (Münster)

Characteristics	STEMI survivors (n = 343)	STEMI non-survivors (n = 67)	p-value (Mann-Whitney/Chi-square)
Age, years, median (IQR)	61.40 (53.8 – 71.3)	66.30 (61.50 – 80.7)	0.0008
Body-mass-index, kg/m^2^, median (IQR)	26.90 (24.69 – 30.40)	27.48 (24.69 – 31.05)	0.90
Male, n (%)	259 (76%)	52 (78%)	0.71
Cardiogenic shock n, (%)	41 (12%)	44 (66%)	< 0.0001
**Risk factors**			
Diabetes mellitus, n (%)	68 (20%)	11 (16%)	0.32
Smoking, n (%)	9 (3%)	1 (1%)	0.16
Hypertension, n (%)	198 (58%)	26 (39%)	< 0.0001
**Patient history**			
Myocardial infarction, n (%)	1 (1%)	0 (0%)	0.66
Atrial fibrillation, n (%)	70 (20%)	17 (25%)	0.39
Stroke, n (%)	3 (1%)	0 (0%)	0.44

**Extended Data Table 3:** Descriptive parameters of the validation cohort (SYSTEMI – Düsseldorf)

Characteristics	STEMI survivors (n = 460)	STEMI non-survivors (n = 51)	p-value (Mann-Whitney/Chi-square)
Age, years, median (IQR)	62.4 (55.8 – 71.5)	76.3 (62.7 – 81.9)	< 0.0001
Body-mass-index, kg/m^2^, median (IQR)	26.2 (24.2 – 29.3)	26.8 (24.1 – 28.3)	0.67
Male, n (%)	353 (77%)	35 (69%)	0.20
Systolic arterial pressure, mmHg, median (IQR)	135 (117 – 157)	115 (96 – 150)	0.0003
Diastolic arterial pressure, mmHg, median (IQR)	80 (69 – 95)	69 (55 – 82)	< 0.0001
Heart rate, bpm, median (IQR)	76 (65 – 91.5)	84 (65.5 – 105)	0.08
Cardiogenic shock n, (%)	56 (12%)	39 (76%)	< 0.0001
**Risk factors**			
Diabetes mellitus, n (%)	170 (37%)	14 (27%)	0.18
Current smoking, n (%)	202 (44%)	10 (20%)	< 0.0001
Hypertension, n (%)	293 (64%)	18 (35%)	< 0.0001
**Patient history**			
Myocardial infarction, n (%)	50 (11%)	4 (8%)	0.50
Percutaneous coronary intervention, n (%)	123 (27%)	18 (35%)	0.19
Atrial fibrillation, n (%)	31 (7%)	6 (12%)	0.19
Stroke, n (%)	13 (3%)	4 (8%)	0.058
Peripheral artery disease, n (%)	30 (7%)	8 (16%)	0.018
