# Supplementary material for: Depth of neutrophil mobilization stratifies survival in ST-elevation myocardial infarction
Source: Nat Cardiovasc Res. 2026 Jul 3;5(8):693–704. doi: 10.1038/s44161-026-00836-0 (PMC13461374; doi:10.1038/s44161-026-00836-0)
Supplement: Supplementary file 1 [file 44161_2026_836_MOESM1_ESM.pdf]

nature cardiovascular research

Supplementary information https://doi.org/10.1038/s44161-026-00836-0

Depth of neutrophil mobilization stratifies survival in ST-elevation myocardial infarction

In the format provided by the authors and unedited

Supplementary Table 1: Aetiologies of the acute chest pain cohort

Patients	Aetiology
9/29	musculoskeletal
3/29	unstable AP with CCS
2/29	hypertensive crisis
2/29	cardiac arrhythmia
2/29	intercostal neuralgia
2/29	psychosomatic
1/29	pulmonary embolism
1/29	pleurisy
7/29	unclear

Supplementary Table 2: Aetiologies of the HFrEF cohort

Patients	Aetiology
31/66	ICM
21/66	DCM
3/66	non-ICM
2/66	TCM
2/66	valvular decompensation
1/66	post-inflammatory
1/66	AS
1/66	HCM
1/66	HFpEF to HFrEF
1/66	non-compaction
1/66	toxic
1/66	restrictive CMP

Supplementary Table 3: Antibodies used for spectral flow cytometry

Antigen	Fluorophore	Clone	Dilution	Vendor	Catalogue#
CD45	Alexa Fluor 700	HI30	1:200	Biolegend	304024
CD8	APC-H7	SK1	1:200	BD Bioscience	560179
CD10	BUV395	HI10a	1:100	BD Bioscience	563871
CD16	BUV496	3G8	1:200	BD Bioscience	612944
CD15	BUV805	W6D3	1:200	BD Bioscience	742057
CD117	BV421	104D2	1:50	Biolegend	313216
CD54	BV480	HA58	1:100	BD Bioscience	746638
CD182	BV650	6C6	1:50	BD Bioscience	744198
CD184	BV785	12G5	1:50	Biolegend	306530
CD66b	FITC	G10F5	1:100	Biolegend	305104
CD4	Pacific Blue	SK3	1:200	Biolegend	344620
CD62L	PE	DREG-56	1:200	Biolegend	304806
CD19	PE- Dazzle594	HIB19	1:200	Biolegend	302252
CD101	PE-Vio770	REA954	1:100	Miltenyi Biotec	130-115-832
CD49d	PE/Cyanine5	REA954	1:100	Biolegend	304306
CD11b	PerCP-Cy5.5	ICRF44	1:50	Biolegend	301328
CD3	Spark Blue 550	SK7	1:100	Biolegend	344852
CD182	APC	5E8	1:50	Biolegend	320710
CD184	APC Cy7	12G5	1:50	Biolegend	306528
CD97	BUV563	VIM3b	1:100	BD Biosciences	748366
CD11a	PE-Dazzle594	HI111	1:100	Biolegend	301231
CD66b	Pacific Blue	G10F5	1:100	Biolegend	305112
