# Supplementary material for: Depth of neutrophil mobilization stratifies survival in ST-elevation myocardial infarction
Source: Nat Cardiovasc Res. 2026 Jul 3;5(8):693–704. doi: 10.1038/s44161-026-00836-0 (PMC13461374; doi:10.1038/s44161-026-00836-0)
Supplement: Supplementary file 2 [file 44161_2026_836_MOESM2_ESM.pdf]

nature portfolio

Corresponding author(s): Oliver Soehnlein & Mathis Richter

Last updated by author(s): 6/06/2026

Reporting Summary

Nature Portfolio wishes to improve the reproducibility of the work that we publish. This form provides structure for consistency and transparency in reporting. For further information on Nature Portfolio policies, see our Editorial Policies and the Editorial Policy Checklist.

Statistics

For all statistical analyses, confirm that the following items are present in the figure legend, table legend, main text, or Methods section.

n/a	Confirmed	
☐	☒	The exact sample size (*n*) for each experimental group/condition, given as a discrete number and unit of measurement
☐	☒	A statement on whether measurements were taken from distinct samples or whether the same sample was measured repeatedly
☐	☒	The statistical test(s) used AND whether they are one- or two-sided<br>*Only common tests should be described solely by name; describe more complex techniques in the Methods section.*
☐	☒	A description of all covariates tested
☐	☒	A description of any assumptions or corrections, such as tests of normality and adjustment for multiple comparisons
☐	☒	A full description of the statistical parameters including central tendency (e.g. means) or other basic estimates (e.g. regression coefficient) AND variation (e.g. standard deviation) or associated estimates of uncertainty (e.g. confidence intervals)
☒	☐	For null hypothesis testing, the test statistic (e.g. *F*, *t*, *r*) with confidence intervals, effect sizes, degrees of freedom and *P* value noted<br>*Give P values as exact values whenever suitable.*
☒	☐	For Bayesian analysis, information on the choice of priors and Markov chain Monte Carlo settings
☒	☐	For hierarchical and complex designs, identification of the appropriate level for tests and full reporting of outcomes
☒	☐	Estimates of effect sizes (e.g. Cohen's *d*, Pearson's *r*), indicating how they were calculated

Our web collection on statistics for biologists contains articles on many of the points above.

Software and code

Policy information about availability of computer code

Data collection	Data acquisition was performed on a Cytek Aurora using SpectroFlo (Cytek Biosciences) software
Data analysis	Data was analyzed using FlowJo v10, Graphpad Prism v10, Excel, ImageJ, R, Python

For manuscripts utilizing custom algorithms or software that are central to the research but not yet described in published literature, software must be made available to editors and reviewers. We strongly encourage code deposition in a community repository (e.g. GitHub). See the Nature Portfolio guidelines for submitting code & software for further information.

Data

Policy information about availability of data

All manuscripts must include a data availability statement. This statement should provide the following information, where applicable:

- Accession codes, unique identifiers, or web links for publicly available datasets
- A description of any restrictions on data availability
- For clinical datasets or third party data, please ensure that the statement adheres to our policy

Source data supporting the findings of this study are provided with this paper.

Research involving human participants, their data, or biological material

Policy information about studies with human participants or human data. See also policy information about sex, gender (identity/presentation), and sexual orientation and race, ethnicity and racism.

Reporting on sex and gender	Information about sex distribution between groups are shown in the supplementary tables
Reporting on race, ethnicity, or other socially relevant groupings	This information has not been collected.
Population characteristics	Informations about population characteristics are shown in the extended data tables.
Recruitment	For flow cytometric analyses, the informed consent of patients in the university hospital Münster which met the inclusion criteria was obtained.<br>For retrospective derivation analysis anonymised patient information including blood counter analysis, clinical chemistry and survival from STEMI patients at the University Hospital Münster between 2019 and 2025 were gathered using a data bank query at the Medical Data Integration Center (MeDIC) of the University Münster with the approval of the Ethics Committee of University of Münster.<br>For the validation cohort, all patients were enrolled in the systemic organ communication in STEMI (SYSTEMI) study (NCT03539133), an open-end prospective cohort study that collects data from STEMI and high-risk non-STEMI patients treated at the University Hospital Duesseldorf. Informed consent was obtained from all enrolled patients included.
Ethics oversight	Ethics Committee of University of Münster and ethics committee of the Heinrich-Heine-University in Duesseldorf, both Germany

Note that full information on the approval of the study protocol must also be provided in the manuscript.

Field-specific reporting

Please select the one below that is the best fit for your research. If you are not sure, read the appropriate sections before making your selection.

☒ Life sciences ☐ Behavioural & social sciences ☐ Ecological, evolutionary & environmental sciences

For a reference copy of the document with all sections, see nature.com/documents/nr-reporting-summary-flat.pdf

Life sciences study design

All studies must disclose on these points even when the disclosure is negative.

Sample size	For flow cytometric analyses:<br>n=37 STEMI patients before revascularization, 11 NSTEMI patients, 29 patients with acute chest pain without troponin dynamics in the emergency department to assess the potential influence of factors not exclusively associated with MI (e.g., fear of death, stress, pain), 21 ischemic stroke patients within the first 24 h after onset of symptoms, 66 patients with heart failure with reduced ejection fraction (HFrEF; LVEF < 40%) and 59 healthy controls<br><br>Retrospective derivation cohort:<br>n = 410 (67 non-survivors)<br><br>Validation cohort:<br>n =511 (51 non-survivors)
Data exclusions	For flow cytometric analyses:<br>Patients with anaemia, acute infectious diseases, acute inflammatory diseases or chronic inflammatory diseases; kidney, liver, haematological or coagulation disorders; malignancies; recent trauma; surgery in the last 6 months; severe bleeding requiring a blood transfusion; hormonal treatment or taking anti-inflammatory drugs, genetic disorders or psychiatric abnormalities; pregnancy and lactation were excluded.
Replication	not applicaple
Randomization	not applicaple
Blinding	not applicaple

Reporting for specific materials, systems and methods

We require information from authors about some types of materials, experimental systems and methods used in many studies. Here, indicate whether each material, system or method listed is relevant to your study. If you are not sure if a list item applies to your research, read the appropriate section before selecting a response.

Materials & experimental systems

n/a	Involved in the study	
☐	☒	Antibodies
☒	☐	Eukaryotic cell lines
☒	☐	Palaeontology and archaeology
☒	☐	Animals and other organisms
☐	☒	Clinical data
☒	☐	Dual use research of concern
☒	☐	Plants

Methods

n/a	Involved in the study	
☒	☐	ChIP-seq
☐	☒	Flow cytometry
☒	☐	MRI-based neuroimaging

Antibodies

Antibodies used

CD45 Alexa Fluor 700 HI30 Biolegend 304024 1:200
CD8 APC-H7 SK1 BD Bioscience 560179 1:200
CD10 BUV395 HI10a BD Bioscience 563871 1:100
CD16 BUV496 3G8 BD Bioscience 612944 1:200
CD15 BUV805 W6D3 BD Bioscience 742057 1:200
CD117 BV421 104D2 Biolegend 313216 1:50
CD54 BV480 HA58 BD Bioscience 746638 1:100
CD14 BV605 M5E2 Biolegend 301834 1:100
CD182 BV650 6C6 BD Bioscience 744198 1:50
CD184 BV785 12G5 Biolegend 306530 1:50
CD66b FITC G10F5 Biolegend 305104 1:100
CD4 Pacific Blue SK3 Biolegend 344620 1:200
CD62L PE DREG-56 Biolegend 304806 1:200
CD19 PE- Dazzle594 HIB19 Biolegend 302252 1:200
CD101 PE-Vio770 REA954 Miltenyi Biotec 130-115-832 1:100
CD49d PE/Cyanine5 REA954 Biolegend 304306 1:100
CD11b PerCP-Cy5.5 ICRF44 Biolegend 301328 1:50
CD3 Spark Blue 550 SK7 Biolegend 344852 1:100
CD182 APC 5E8 Biolegend 320710 1:50
CD184 APC Cy7 12G5 Biolegend 306528 1:50
CD97 BUV563 VIM3b BD Biosciences 748366 1:100
CD11a PE-Dazzle594 HI111 Biolegend 301231 1:100
CD66b Pacific Blue G10F5 Biolegend 305112 1:100
CD66b FITC, Miltenyi Biotec 1:50
CD10 APC, Miltenyi Biotec 1:50
CD16 PerCP, Miltenyi Biotec 1:50

Validation

All antibodies are commercially available and were validated by the vendors.

Clinical data

Policy information about clinical studies
All manuscripts should comply with the ICMJE guidelines for publication of clinical research and a completed CONSORT checklist must be included with all submissions.

Clinical trial registration

Ethics for flow cytometric analyses and retrospective data analysis:2021-424-f-S, 2021-532-f-S, 2023-348-f-S, 2023-489-f-S, 2024-185-f-S and 2024-253-f-S (University of Münster)
Prospective open-end prospective cohort study NCT03539133 #5961R (University Hospital Düsseldorf)

Study protocol

Prospective open-end prospective cohort study NCT03539133 #5961R (University Hospital Düsseldorf): 10.1186/s12872-023-03210-1

Data collection

Flow cytometric analyses: University Hospital Münster (2021-2024)
Retrospective derivation cohort: 2019-2024
Validation cohort: 2019-2023

Outcomes

Death within 30 days after STEMI

Plants

Seed stocks

Report on the source of all seed stocks or other plant material used. If applicable, state the seed stock centre and catalogue number. If plant specimens were collected from the field, describe the collection location, date and sampling procedures.

Novel plant genotypes

Describe the methods by which all novel plant genotypes were produced. This includes those generated by transgenic approaches, gene editing, chemical/radiation-based mutagenesis and hybridization. For transgenic lines, describe the transformation method, the number of independent lines analyzed and the generation upon which experiments were performed. For gene-edited lines, describe the editor used, the endogenous sequence targeted for editing, the targeting guide RNA sequence (if applicable) and how the editor was applied.

Authentication

Describe any authentication procedures for each seed stock used or novel genotype generated. Describe any experiments used to assess the effect of a mutation and, where applicable, how potential secondary effects (e.g. second site T-DNA insertions, mosiacism, off-target gene editing) were examined.

Flow Cytometry

Plots

Confirm that:

☒ The axis labels state the marker and fluorochrome used (e.g. CD4-FITC).

☒ The axis scales are clearly visible. Include numbers along axes only for bottom left plot of group (a 'group' is an analysis of identical markers).

☒ All plots are contour plots with outliers or pseudocolor plots.

☒ A numerical value for number of cells or percentage (with statistics) is provided.

Methodology

Sample preparation

For flow cytometric analysis of leukocytes, EDTA blood was incubated with red blood cell lysis buffer (Biolegend) for 20 minutes on ice for erythrocyte lysis. After washing, cells were centrifuged and resuspended in HANKs buffer (HBSS w/o Mg2+ and Ca2+, 0.06% BSA, 0.3mM EDTA) and unwanted FcR-involved staining blocked using Human TruStain FcX (Biolegend). Afterwards, cells were incubated with fluorophore-conjugated antibodies for 20 min on ice in Cell Staining buffer (Biolegend) prior to measurement.

Instrument

5L-Cytek® Aurora (Cytek Biosciences)

Software

SpectroFlo, FlowJo v10.10.0 (BD Bioscience), Rstudio and GraphPad Prism 10 (GraphPad Software, USA)

Cell population abundance

Describe the abundance of the relevant cell populations within post-sort fractions, providing details on the purity of the samples and how it was determined.

Gating strategy

Doublets were excluded using forward and side scatter characteristics and dead cells using DAPI. Neutrophils were identified as CD66b+SSChighCD49dlow cells, eosinophils as CD66b+CD16low cells, B cells as CD66b-CD19+CD3- cells, T cells as CD66b-CD19-CD3+ cells and further divided into CD4+ and CD8+ T cells while monocytes were defined as CD66b-CD19-CD3-SSCintFSChigh cells

☒ Tick this box to confirm that a figure exemplifying the gating strategy is provided in the Supplementary Information.
